# The Biochemical Properties and Functions of CALM and AP180 in Clathrin Mediated Endocytosis

**DOI:** 10.3390/membranes4030388

**Published:** 2014-07-31

**Authors:** Lia Moshkanbaryans, Ling-Shan Chan, Mark E. Graham

**Affiliations:** Children’s Medical Research Institute, The University of Sydney, 214 Hawkesbury Road, Westmead, NSW 2145, Australia; E-Mails: lmoshkanbaryans@cmri.org.au (L.M.); lingshan.chan@gmail.com (L.-S.C.)

**Keywords:** CALM, phosphatidylinositol binding clathrin assembly protein, AP180, clathrin, adapter protein complex 2, endocytosis, vesicle, clathrin assembly, cargo sorting, AP180 N-terminal homology domain, phosphorylation, O-GlcNAc-6-phosphate

## Abstract

Clathrin-mediated endocytosis (CME) is a fundamental process for the regulated internalization of transmembrane cargo and ligands via the formation of vesicles using a clathrin coat. A vesicle coat is initially created at the plasma membrane by clathrin assembly into a lattice, while a specific cargo sorting process selects and concentrates proteins for inclusion in the new vesicle. Vesicles formed via CME traffic to different parts of the cell and fuse with target membranes to deliver cargo. Both clathrin assembly and cargo sorting functions are features of the two gene family consisting of assembly protein 180 kDa (AP180) and clathrin assembly lymphoid myeloid leukemia protein (CALM). In this review, we compare the primary structure and domain organization of CALM and AP180 and relate these properties to known functions and roles in CME and disease.

## 1. Introduction

Clathrin mediated endocytosis (CME) is a fundamental multi-functional biological process. These functions include the internalization of receptors, recycling of membrane components, internalization of toxins and viruses, nutrient uptake and activation of signaling pathways including those controlling development and immune responses (for a review of CME, see [1,2]). In CME, cargo bearing clathrin coated vesicles are created *de novo* at the plasma membrane (PM), internalized and then traffic either back to the surface or to early endosomes, which act as traffic way-stations, then to other intracellular locations such as recycling endosomes, multi-vesicular bodies and lysosomes [1]. Vesicles produced by CME or synaptic vesicle endocytosis (SVE) [3] must include proteins for targeting and fusion of the vesicle with the correct intracellular membranes [4]. Thus, how vesicles are made and what cargo is included has major downstream effects on broad cellular functions and may contribute to disease states.

CME is the production of a vesicle using a clathrin coat and many adapter and accessory proteins that are evolutionarily conserved in different cell types and organisms [1,3]. SVE is a specialized version of CME that occurs in neurons to produce synaptic vesicles (SVs). SVE uses some brain specific homologs of the ubiquitously expressed CME protein machinery. A major difference between vesicles made by CME and SVE is that SVs are smaller in diameter, which is likely due to mechanistic differences in how adapter proteins sort cargo and assemble the clathrin coat [1,5]. We will discuss the genetically related proteins CALM and AP180, which have roles in both cargo sorting and assembly in CME and SVE. In particular, we will relate their sequence similarities and differences to their known functions.

## 2. The Early Stages of CME

**Figure 1:**
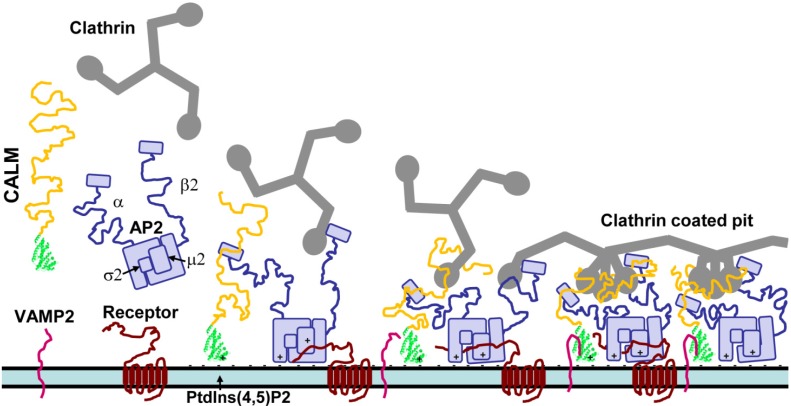
The interactions of CALM during the early stages of CME.

The first stage of CME is initiation, followed by formation of a clathrin coated pit and cargo selection [1,2] (Figure 1). The exact mechanism of initiation of CME is not yet fully understood [6], although the key components are well known. Clathrin has no lipid binding ability on its own [7,8,9], so must be recruited to nascent sites of endocytosis by adapters that can bind both clathrin and lipid. Accessory proteins that bind adaptors and have other endocytic functions, such as membrane remodeling, may also be present during initiation or soon after. Rather than an ordered event, initiation might be achieved by different combinations of protein components that associate stochastically [6,9]. An essential component for initiation is a high local concentration of phosphatidylinositol (4,5)-bisphosphate [PtdIns(4,5)P2] in the PM to define the site of adapter and clathrin nucleation [9].

The interactions of clathrin and adapter proteins during the early stages of CME is shown in Figure 1. CALM and adaptor protein complex 2 (AP2) accumulate at sites of enriched PtdIns(4,5)P2, via binding of specific basic sequences. CALM interacts directly with the α subunit of AP2. Cytosolic clathrin is recruited by the β2 subunit of AP2. CALM may also have a role in recruitment but this has not been proven. CALM and AP2 co-assemble clathrin using clathrin binding motifs in disordered domains to form a coated pit. AP2 binds receptor cargo. The CALM AP180 N-terminal homology (ANTH) domain (represented by the crystal structure from Protein Data Bank entry 3ZYK in Figure 1) binds the soluble NSF (N-ethylmaleimide-sensitive fusion protein) attachment protein receptor (SNARE) domain of vesicle associated membrane proteins (VAMPs) [10,11]. The knockout/depletion phenotype of CALM suggests that it has a role in ordering and tightening the clathrin cage [12,13,14,15]. The interactions and role of AP180 in CME is essentially the same as CALM in Figure 1, except that AP180 has a longer disordered chain with more clathrin binding motifs that might be responsible for producing smaller clathrin coated vesicles.

The ubiquitous adaptor protein complex 2 (AP2) is a heterotetramer of four subunits (α, β2, µ2 and σ2 [16]) and is specific for CME at the PM. AP2 binds to PtdIns(4,5)P2 via binding sites on the α and µ2 subunits [17] and is able to recruit clathrin triskelia via the β2 subunit and drive clathrin assembly [9,18,19] (Figure 1). Both α and β2 subunits can bind clathrin, but also a wide range of adapter and accessory proteins, e.g., CALM and AP180, epidermal growth factor receptor substrate 15 (eps15), epsins, amphiphysins and intersectins [8,20,21]. The µ2 subunit recognizes protein cargo binding motifs [8,16,22]. In this way, AP2 is a major protein interaction hub due to its ability to recruit numerous transmembrane, adaptor and accessory proteins to CME nucleation points, while binding to clathrin [23,24]. Initiation often involves recruitment of one clathrin triskelion by two AP2 complexes [9]. Fully formed clathrin coated vesicles (CCVs) contain a reduced ratio of one clathrin triskelion for each AP2 complex, but AP2 is still the equal most abundant CCV-associated protein [25].

**Figure 2:**
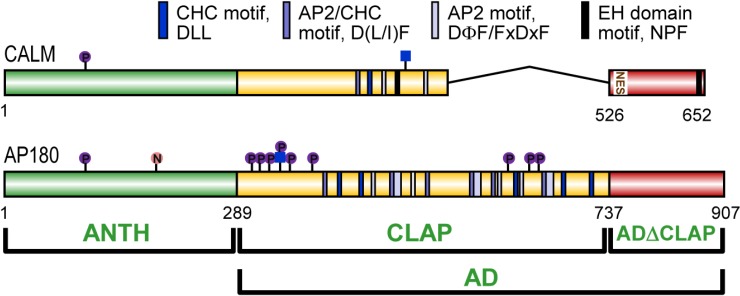
Domain structure of human CALM and AP180. AP180 consists of an AP180 N-terminal homology (ANTH) domain and an assembly domain (AD). The AD has two subdomains: a clathrin and adapter (CLAP) domain and the remainder is an unnamed domain (ADΔCLAP). CALM has a nuclear export signal (NES). AP180 is nitrated (“N” in orange circle), phosphorylated (“P” in purple circle) and O-GlcNAc-6-phosphate modified (blue square with “P” in purple circle above). CALM is phosphorylated and O-GlcNAc modified (blue square only). Clathrin heavy chain (CHC), AP2 and eps15 homology (EH) domain binding motifs are shown.

The other major adaptor proteins are CALM and AP180, which are equally abundant with AP2 in CCVs (in CME and SVE, respectively) at a ratio of one adapter molecule per clathrin triskelion [25,26], but have often been overlooked for detailed study of their role in CME/SVE. CALM and AP180 are monomeric adapter proteins that bind to the PM via their N-terminal ANTH domain [27] and bind clathrin and AP2 via an unstructured C-terminal assembly domain (AD) [28] (Figure 1 and Figure 2). Many other less abundant monomeric adapters have a similar domain structure that allows for lipid, clathrin, cargo and accessory protein binding [29]. The arrival of AP2 during initiation has been well defined using single molecule resolution microscopy of CME, but this does not rule out a role for monomeric adapters in initiation [9]. Another high resolution imaging study demonstrated that clathrin and CALM have the same profile of localization during CME [30], indicating co-recruitment. An *in vitro* study showed that AP180 can recruit clathrin to synthetic lipid monolayers and that the monomeric adapter, epsin, could assemble a clathrin lattice and drive budding of CCVs [31]. This suggests that AP2 might be redundant in a subset of CME events. CALM and epsin, but not AP2, are required for CME of notch ligands [32]. However, knockdown experiments have argued against an essential role of AP180/CALM in the early stages of CME. When CALM is knocked down, there are still clathrin coated pits at the PM, but pits are greatly depleted when AP-2 is knocked down [19]. There are many PM localized monomeric adapters that could potentially compensate for some of the lost functions of CALM or AP180 knockdown, but it is likely more difficult to replace the AP2 interaction hub function at the PM since the other AP complexes are localized to organelle membranes [1].

Maturation of clathrin coated pits requires interaction of AP2 with adapter and accessory proteins via its α subunit [33]. Clathrin, adapter and accessory proteins bind to the α and β2 subunit appendage domains with differing affinities [21,24]. Other adapters and accessory proteins with BAR (bin-amphiphysin-rvs) domains are able to sense lipids with different curvature and induce curvature [34]. The globular N-terminal domain of clathrin heavy chain has multiple binding sites for adapter proteins, allowing influence over clathrin coated pit dynamics [35]. Arising from this knowledge is a model where sensing/inducing lipid curvature and affinity for AP2 and clathrin defines how adapter and accessory proteins are spatially and temporally recruited and localized to fulfill their specific role in CME. This spatiotemporal regulation must also ensure that cargo selection occurs prior to budding. CALM andAP180 ensure that VAMPs/synaptobrevins are sorted into nascent vesicles by direct binding to the ANTH domain [10,11]. Thus, CALM and AP180 are abundant CME proteins with domains and sequence motifs that bind lipid, cargo, AP2 and clathrin, which predict roles in initiation, CME protein nucleation, cargo selection and clathrin coat assembly, but not all of these potential roles have been proven or fully investigated.

## 3. CALM and AP180 Domain Structure and Sequence Similarities

CALM and AP180 have two domains with known functions; a folded ANTH domain, which binds PtdIns(4,5)P2 and VAMPs; and a C-terminal assembly domain (AD), which has no known secondary structure (Figure 2).

### 3.1. A Structured ANTH Domain

In 2001, two groups reported the ANTH domain crystal structure, one using the sequence from the *Drosophila melanogaster* homolog, LAP (like-AP180) [36] and the other using *Rattus norvegicus* CALM [37]. They showed that the ANTH domain is a globular structure consisting of ten folded alpha helices and is similar to the smaller epsin N-terminal homology domain. A lysine in α1 and a KKKH sequence in helix α2 bind PtdIns(4,5)P2 [36,37]. The ANTH domain in mammalian AP180 sequences is highly homologous (Figure 2). The rat, mouse and human ANTH (1-289) share >98.9% identity; in CALM the three mammalian sequences are 100% identical (Figure 2, aligned using ClustalW2. See also [38]). A comparison of AP180 and CALM ANTH domains within each of these mammals indicates an average of 81.4% identity. The identity drops when comparing the human CALM and AP180 ANTH domains to that of LAP (69.6% and 67.1%), the *Caenorhabditis elegans* homolog uncoordinated protein 11 (Unc11, 65.1% and 63.0%) or the *Saccharomyces cerevisiae* homolog yeast AP180A (YAP180A, 23.7% and 23.7%). Despite low identity with mammalian CALM and AP180, the role of the ANTH in VAMP internalization [10,11,39] seems to be evolutionarily conserved, since YAP180 has a role in the cargo-specific internalization of the yeast VAMP2 homolog, Snc1 [40]. YAP180A and YAP180B are coded by different yeast genes and are functionally redundant in sorting Snc1. The homolog of CALM and AP180 in *Dictyostelium discoideum* has also been shown to sort VAMP7B into clathrin coated vesicles on contractile vacuoles [41].

### 3.2. A Disordered Assembly Domain with Short Protein Binding Motifs

The sequence of the AP180 AD has been studied in the most detail. The AP180 AD is a disordered random coil which occupies the same space as would a much larger globular protein [42]. The physical properties of the AD account for the slower migration of the full length protein on SDS-PAGE gels [43] at an apparent 180 kDa, despite its molecular mass of 92.5 kDa. CALM and AP180 AD sequences are not as evolutionarily conserved as the ANTH domain. The human, rat and mouse CALM and AP180 ADs have high average identities (96.0% and 90.3%, respectively). However, the AD of other organisms are generally shorter. The AD of LAP is 54% and 73% shorter than human CALM and AP180. Unc11 is 23% and 55% shorter and YAP180A is 42% and 1% shorter. Thus, non-mammalian AD identities are low. By comparison with human CALM and AP180, LAP is 17.0% and 23.6% identical, Unc11 is 11.8% and 16.43% identical and YAP180A is 12.4% and 12.1% identical. This lack of identity does not prevent the various ADs from performing functions involving clathrin and AP2 binding, since these interaction rely on the presence of short binding motifs [20,28], but likely reflects differing requirements and specialization of CME in different organisms and tissues. A subset of CALM and AP180 homologs also have NPF motifs, which bind eps15 homology domains [44]. Mammalian AP180 harbors the most clathrin and AP2 binding motifs, but has no NPF motifs.

**Figure 3:**
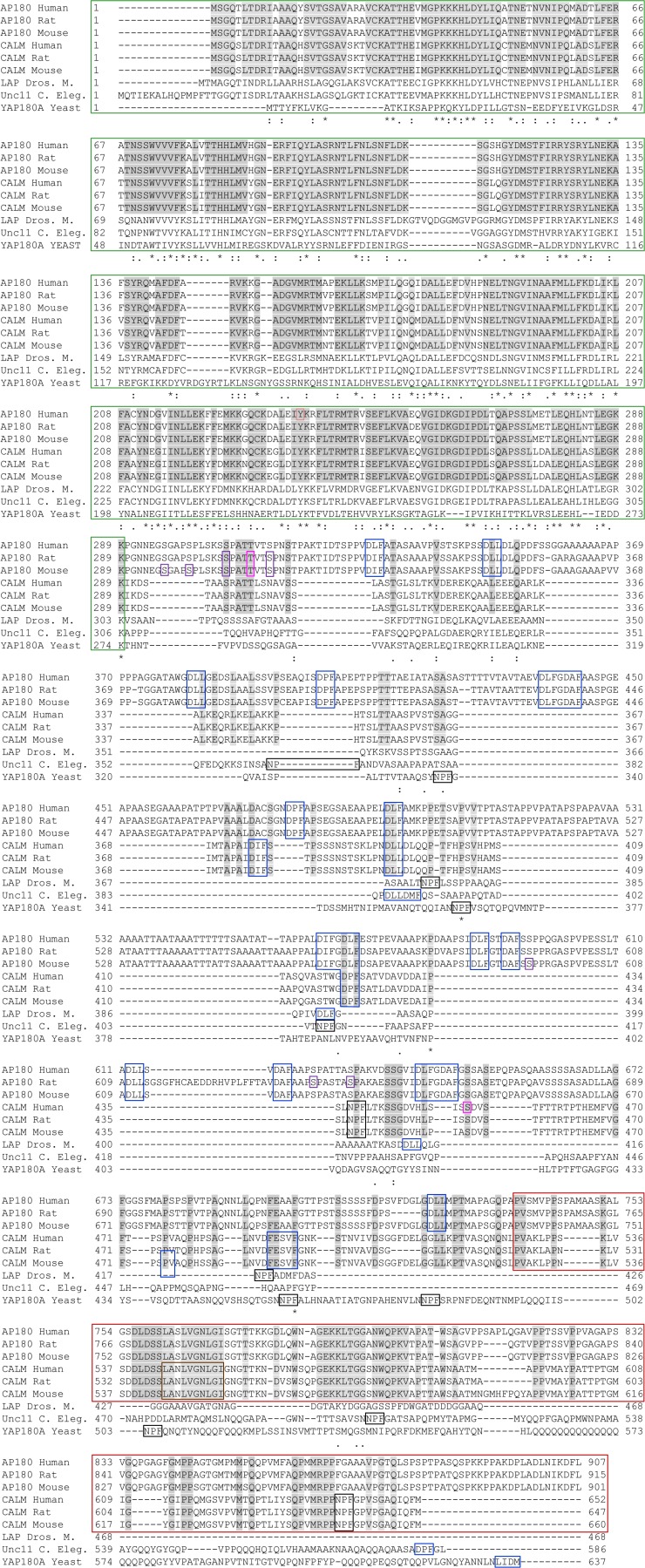
Alignment of isoform 1 of CALM and AP180 family members. Uniprot accessions: O60641, Q05140, Q61548, Q13492, Q498N4, Q7M6Y3, Q9VI75, Q9XZI6 and P38856. Domains, motifs and post-translational modifications are boxed using Figure 2 color scheme, except that all CLAP motifs are blue and O-GlcNAc-P (AP180 only) and O-GlcNAc (CALM) are pink. Identical residues in mammalian CALM and AP180 are shaded grey. Similarity across commonly researched organisms are indicated below: “*”, identical; “:”, strong similarity; “.”, weak similarity. Only phosphosites listed at PhosphoSite.org with ≥3 journal article references, or manually verified by our group [45] are shown.

The clathrin and AP2 motifs in mammalian CALM and AP180 do not extend all the way to the C-terminus and thus form a central clathrin and adaptor protein binding (CLAP) sub-domain (Figure 2 and Figure 3). A three domain structure is supported by isoelectric point analysis of rat [46] and mouse [47] AP180 domains. The CLAP domain is acidic (human AP180 290-729; pI 3.2; human CALM 290-525, pI 5.2, our results using [48]. Sequence alignment does not support a central CLAP domain for LAP, Unc11 or YAP180, which have CLAP motifs that extend to near or at the extreme C-terminus [49] (Figure 3, aligned using ClustalW2 [50], settings were default except that five tree iterations were done). Nevertheless, the ADs of these homologs are acidic (pI 3.7, 6.0 and 5.8, respectively). In contrast, the isoelectric points of the ANTH domains of CALM, AP180 and homologs are basic: human AP180, 9.1; human CALM, 9.3; LAP, 8.5; Unc11, 8.1; and YAP180A, 9.1. The remaining C-terminal tail of the mammalian CALM and AP180 ADs, which we are calling the ADΔCLAP, is also basic. The human CALM and AP180 ADΔCLAPs have a pI of 9.5 and 10.2. The ADΔCLAP has no specific function in CME, but was recently found to harbor a nuclear export signal (NES) in CALM [51,52] (human CALM, 544-LANLVGNLGI-533). The NES consensus sequence is ΦX_1-3_ΦX_2-3_ΦXΦ, where Φ is most often leucine. The CALM NES appears to be a conserved sequence in mammalian CALM and AP180 (Figure 3). Evidence for nucleocytoplasmic shuttling of epsin, eps15 and CALM was found over a decade ago but the role of these endocytic proteins in the nucleus is unknown [53]. Also, CME proteins have a role in mitosis which is independent of endocytosis [54]. CALM depleted HeLa cells have increased multinucleation and show delayed formation of the cleavage furrow. In summary, each CALM or AP180 homolog has short binding motifs for clathrin and adapter/accessory protein binding and mammalian CALM and AP180 appear to have a three domain structure which includes a central acidic CLAP domain.

## 4. The Function of the CALM and AP180 Assembly Domain

### 4.1. Clathrin Binding and Assembly

A major tool for determining the role of the AD has been the use of truncated recombinant CALM and AP180 to compare each fragment’s ability to bind proteins and assemble clathrin, *in vitro*. The recombinant AP180 AD was found to be the minimum sequence required to assemble clathrin cages *in vitro* as efficiently and rapidly as the full length sequence [28,55]. A 42 kDa AP180 fragment, similar to the CLAP domain, could bind triskelia and cages but had no assembly function [55]. A 16 kDa C-terminal fragment bound clathrin cages, but had no assembly function [55], unless high concentrations were used in the assay [28]. Therefore, the entire AD of AP180, *i.e.*, the CLAP plus the ADΔCLAP, is required for efficient clathrin assembly function.

Full length CALM assembles clathrin cages *in vitro* [56,57]. Truncated CALM sequences have not been tested for assembly activity, but have been used to assess their ability to bind clathrin. These fragments do not correlate with the three domain structure we have proposed for CALM (Figure 2). Nevertheless, a similar pattern has emerged. C-terminal fragments approximating the AD, human CALM 414-652 [58] or 256-652 [59], can bind clathrin similar to the full length sequence, but sequences with a truncated C-terminus, *i.e.*, 1-413, 414-613 [58] or 256-583 [59] bind clathrin poorly. This indicates that, similar to AP180, the entire AD of CALM is required for efficient clathrin binding. These *in vitro* observations are supported by transferrin (Tfn) uptake assays [58,59]. Full length GFP-CALM has a dominant negative effect when transfected, partially blocking CME. GFP fusions with most of the AD plus an intact C-terminus similarly block Tfn uptake, but C-terminally truncated CALM has reduced or no effect on Tfn uptake [58,59]. The effect of partial AD truncation on Tfn uptake has not been tested for AP180. Thus, the entire AD is required for efficient clathrin binding by CALM *in vitro* and *in vivo*.

The CALM and AP180 assembly functions are required for the production of small uniform vesicles, in CME and SVE, respectively, as shown in a number of studies [12,13,60,61,62]. Several groups showed by electron microscopy that purified or bacterially expressed mammalian AP180 assembled clathrin into cages of narrower diameter compared to when AP180 was absent [60,61,62]. These *in vitro* assays highlighted that a modest variation in SV diameter would lead to a large difference in SV volume and neurotransmitter content. The quantal theory of neurotransmitter packaging reflects the observation that SV size is tightly regulated. Thus, it has been proposed that AP180 and homologs are crucial for SV size regulation [61]. This proposal has been confirmed by *in vivo* observations of cells lacking LAP [12] and Unc11 [13], which are present in all cells of *D. Melanogaster* and *C. Elegans*, respectively, but are also enriched in nerve terminals and have a synaptic phenotype. Functional knockout of *lap* and *Unc11* resulted in an increase in SV size and quantal neurotransmitter release [12,13]. Additionally clathrin and VAMP2 were mis-localized, synapses failed to produce a sufficient number of SVs and the probability of neurotransmitter release was reduced [12,13]. Depletion of CALM [14] or AP180 [15] also results in abnormally large and deformed vesicles. *VAMP2* knockout has a similar phenotype to knockout of CALM and AP180, including a change in SV size and shape [63]. This, and the observation that knockout of other SV cycle proteins also affect vesicle size [64], led to uncertainty surrounding the role of the CALM and AP180 AD in shaping vesicles. However, it has recently been shown, by ablation of the VAMP2 sorting function, that the regulation of vesicle size and shape by CALM is independent of its VAMP sorting function [65], *i.e.* it is a clathrin assembly defect. Thus, CALM and AP180 have an evolutionarily conserved role in the high fidelity production of vesicles with a consistent size and shape.

### 4.2. Mechanism of Clathrin Assembly

The identification of multiple short clathrin binding motifs in the AD of AP180 provided a structural basis for the ability of CALM and AP180 to rapidly assemble clathrin [28,66]. AP180 contains eleven D(L/I)(L/F) motifs (Figure 2 and Figure 3), more than any other CME adapter protein, which may account for its ability to assemble clathrin *in vitro* four times faster than AP2 [67]. These same motifs are found in CALM, LAP, Unc11, other homologs, other CME adapters [28] and the clathrin cage disassembly protein, auxilin [68]. A high concentration of a D(L/I)(L/F) motif peptide was shown to block endocytosis [28]. AP180 binds the clathrin heavy chain (CHC) terminal domain (TD) [28] and these short motifs are expected to bind to similar grooves that clathrin box motifs use to bind the TD [69,70], but the D(L/I)(L/F) motif TD binding site is not known. A high number of D(L/I)(L/F) motifs in AP180 may explain its high clathrin assembly activity. A linear relationship between clathrin assembly and the number of D(L/I)(L/F) motifs was established in AP180 by progressive N-terminal truncation [28]. Multiple motifs may also explain how a tighter clathrin cage is achieved in the production of SVs. AP180 D(L/I)(L/F) motifs are hypothesized to cross-link multiple clathrin TDs [28].

More recently, a nuclear magnetic resonance experiment using the CHC TD and a fragment of AP180 containing two D(L/I)(L/F) motifs revealed that these motifs have weak affinity (K_d_~250 µM), fast on and off rates and have localized β-turn-like structures that do not change in the free or bound state [66]. Apart from the detailed study of these two motifs, systematic analysis of the contribution of AP180 clathrin binding motifs to AP180 function has not been done. The current knowledge on AP180-clathrin binding motifs has led to the line of baited hooks model [42,66,71], *i.e.*, multiple D(L/I)(L/F) motif “hooks” along the AD “line” of AP180 are free to “fish” for clathrin to allow dynamic recruitment and assembly. AP180 likely co-assembles clathrin with AP2, since AP180 also has multiple binding motifs for the AP2 α subunit (Figure 2) and an AP180-AP2 complex has been shown to assemble clathrin cages *in vitro* faster than either protein alone [72].

CALM has only a single DLL motif. A DIF motif could potentially bind clathrin, but this motif is also one of three sites shown to bind the AP2 α subunit [14]. Nevertheless, *in vitro* peptide affinity experiments indicate that clathrin and AP2 can potentially bind to a quite broad set of motifs and share these short binding motifs [68]. Therefore, CALM may appear underequipped for multivalent binding to clathrin, but there may be additional unidentified clathrin binding sites. Two studies have shown that CALM C-terminal sequences that do not contain the DLL motif are involved in clathrin binding and influence receptor uptake assays [58,59]. This indicates there are at least two clathrin binding sites in CALM.

The longest isoform of human CALM has two NPF motifs (Figure 2), but no binding to EH domain proteins eps15 or eps15R has been observed [58]. However, squid AP180 binds eps15 and may stimulate clathrin assembly [44]. YAP180A and B have five NPF motifs and knockout studies in yeast suggest that adaptor-accessory protein (*i.e.*, NPF-EH domain) interactions regulate the transition from early to late endocytic events [73].

## 5. The Cargo Sorting Function of the CALM and AP180 ANTH Domains

Different types of cargo are recognized by various CME adapter proteins that concentrate cargo within clathrin coated pits to ensure their internalization [74]. The CALM and AP180 ANTH domains bind to cargo proteins in the VAMP family [10,11,39,65,75]. VAMP is an essential component of the SNARE complex, along with other core components: synaptosomal associated protein 25 (SNAP25) and syntaxin [76,77]. CALM and AP180 bind directly to VAMP2, 3 and 8 and sort them into vesicles at the PM [10,11] and this role has been recently reviewed [75,78]. More recently, it has been shown that CALM also has the potential to sort VAMP4 and 8, indicating the universality of CALM-VAMP interactions [65].

Since VAMP2 is the most abundant synaptic vesicle protein component [79] and is required for fast calcium triggered SV fusion [80], there is a strong synaptic phenotype following CALM or AP180 knockout/depletion. *Lap* knockout results in reduced neurotransmitter release [12], disrupted calcium coupling to exocytosis and mis-localization of a VAMP2 homolog [81]. Likewise, *Unc11* knockout mis-localizes a VAMP2 homolog and reduces neurotransmitter release [13]. More recently, LAP function was studied using a system which allowed acute photoinactivation of a *lap* transgene [82]. It was shown that acute *lap* knockout had no effect on exocytosis, but was required for endocytosis and maintenance of the protein composition of SVs. Mammalian CALM knockdown was shown to affect VAMP2 surface levels in HEK293 cells [11,39], PC12 cells [39], HelaM cells [10] and hippocampal neurons [11].

Structural studies identified specific CALM-VAMP interaction sites. CALM does not use short linear motifs to recognize cargo [4,74], but binds VAMPs via their SNARE domains [10,11,83], the same domain VAMPs use to form four-helical bundles with other SNAREs to drive membrane fusion [77]. VAMPs bind in a long groove on the ANTH domain surface and can bind PtdIns(4,5)P2 simultaneously to increase ANTH affinity for membranes [10]. SNARE complex formation and ANTH binding was shown to be mutually exclusive for VAMP8. Knowledge of the residues involved in VAMP binding has provided tools to study the VAMP sorting function of CALM and AP180 independently of the clathrin assembly function, e.g., the M244K mutant does not bind VAMPs [10,65]. Thus CALM and AP180 load VAMP into vesicles, ensuring their competence for future SNARE mediated membrane fusion.

Evidence for direct binding of AP180 to VAMPs is weaker than for CALM. One study showed that GST-AP180 ANTH could extract recombinant VAMP2 from solution better than GST, but with a greatly reduced ability in comparison to CALM [11]. Another study found no evidence of direct AP180-VAMP interaction [10]. This is surprising, since the CALM and AP180 ANTH domain sequences have few non-identical/similar residues (Figure 3), but may hint at differences in the VAMP sorting mechanism or different roles of CALM and AP180 in neurons.

## 6. Role of CALM in Receptor Uptake

Overexpression of CALM or AP180 blocks uptake of standard cargoes, *i.e.*, Tfn and the epidermal growth factor receptor (EGFR), via CME by dominant negative sequestration of clathrin [37,58,59,84]. The AD of AP180 has been used as a tool to block CME in dozens of studies (e.g., see [85]), although one study reported no change in Tfn uptake [86]. However, depletion of CALM has little or no effect on uptake of Tfn or EGFR. In early studies, CALM depletion appeared to have no effect on Tfn uptake, but a specific effect on EGFR uptake [14,87]. Two later studies confirmed that CALM depletion had no effect on Tfn uptake [10,39]. A small but non-significant effect on EGF uptake was observed in a recent study [10]. In a different type of experiment, Tfn uptake was affected in CALM knockout mouse embryonic fibroblasts [88] and CALM-deficient mice suffered growth retardation and severe anemia. This highlights the difficulty in establishing the role of CALM in receptor uptake when using approaches that allow cells to potentially adapt and compensate after CALM depletion.

## 7. Post-Translational Modifications of CALM and AP180

AP180 and other SVE adapters and accessory proteins, are known to be phosphorylated in unstimulated nerve terminals then undergo coordinated dephosphorylation by the calcium stimulated phosphatase calcineurin [89]. The model for this paradigm is endocytosis fission protein dynamin 1, which is required to be dephosphorylated at particular phosphorylation sites to allow dynamin 1-protein interactions that promote endocytosis [90,91]. Thus, dephosphorylation of AP180 is likely to promote AP180-protein interactions and SVE. This has not been shown, but comparison of native and recombinant AP180 has demonstrated that modification of AP180 is not required for the clathrin assembly function [61,92]. AP180-AP2 binding and cooperative clathrin assembly is weakened when AP180 is phosphorylated by casein kinase 2 *in vitro* [72].

Using ^32^P metabolic labeling, it was found that AP180 is relatively heavily phosphorylated on predominantly serine residues [93,94] and phosphorylation occurs mainly in the AD [92]. Many phosphorylation sites have been identified on AP180 in both targeted [45] and large scale phosphoproteomics studies [95,96,97,98,99] (Figure 2 and Figure 3). None of the published AD *in vivo* phosphorylation sites match the casein kinase 2 substrate consensus sequence, (S/T)*X_n_*(D/E) where n is often three but also one or two [100]. However, in the acidic AD there are peptides produced from trypsin cleavage of AP180 that contain multiple potential casein kinase 2 sites but are >10 kDa and not amenable to conventional phosphorylation site analysis. The use of small molecule protein kinase inhibitors on synaptosomes has ruled out protein kinase C and cyclin-dependent kinase 5 as major protein kinases for AP180 [90,101]. DYRK1A is able to phosphorylate AP180 to dissociate it from clathrin coated vesicles in an *in vitro* assay [102], but there is no evidence that this occurs *in vivo*. Thus, the *in vivo* protein kinases that phosphorylate AP180 remain to be discovered and the functions of the known AP180 phosphorylation sites in SVE are yet to be determined.

AP180 is also a glycoprotein. AP180 was the first known protein to be modified by the O-GlcNAc-6-phosphate modification [45]. Other synaptic proteins also contain this modification [103]. O-GlcNAc and O-GlcNAc-6-phosphate have been found at the same site in AP180 (Thr-310, Figure 2). Thus, these modifications have the potential to dynamically regulate AP180 protein interactions at the phosphorylation and glycosylation levels in perhaps a stepwise manner [104]. CALM is also modified by O-GlcNAc [105,106] and phosphorylated at multiple sites [107]. Since very few of these site localizations have been independently verified, we have included only those repeatedly or confidently identified in Figure 2. A phosphorylation site detected at Ser107 in both neuronal and non-neuronal cells could be from either CALM or AP180 since the tryptic peptide sequence is identical. AP180 purified from the human pituitary was found to be modified by nitration at Tyr237 [108]. Both CALM and AP180 are also subject to additional types of modification, e.g., ubiquitination and di-methylation [107,109,110]. The roles of these post-translational modifications in regulating CALM and AP180 functions remain to be determined.

## 8. CALM and AP180 in Disease

### 8.1. Leukemia

CALM was discovered as product of a t(10;11)(p13;q14) translocation in the U937 cell line, derived from a diffuse histiocytic lymphoma [111,112]. This translocation gives rise to the *PICALM-MLLT10* fusion gene and CALM-AF10 fusion protein, which is found in patients with acute myeloid leukemia, T-cell acute lymphoblastic leukemia and malignant lymphoma [113,114,115]. Multiple breakpoints have been identified, but the major protein product is always a mildly C-terminally truncated CALM fused to a mildly N-terminally truncated AF10. Recent studies have shed light on the mechanism of CALM-AF10 mediated leukemogenesis. AF10 interacts with DOT1L, a methyltransferase that methylates H3K79 (histone H3 lysine 79), through its OM-LZ (octapeptide motif–leucine zipper) interaction domain. H3K79 hypermethylation upregulates *HOXA* gene clusters, which is critical for CALM-AF10 induced leukemogenesis [52,116]. It was later demonstrated that direct inhibition of the methyltransferase activity of DOT1L via genetic inactivation and small molecule inhibition prevented CALM-AF10 mediated leukemogenesis [117].

The CALM contribution to leukemogenesis from the CALM-AF10 protein was independently discovered by two laboratories and attributed to the presence of an NES (Figure 2 and Figure 3) in CALM [51,52]. The CALM NES fused to AF10 was necessary and sufficient for immortalization of cells *in vitro* and to induce leukemia in mice [51,52]. However, it is not clear how cytoplasmic localization or nucleocytoplasmic shuttling of CALM-AF10 induces leukemia, since DOT1L is only partially mis-localized by CALM-AF10 [51], if at all [52]. It is also not known how the endocytic function of CALM impacts leukemogenesis. CALM-AF10 expression was associated with altered Tfn uptake efficiency in 293T cells; however, endocytosis and proliferation were unaffected in a leukemia cell line [118].

### 8.2. Alzheimer’s Disease

The gene for CALM, *PICALM*, was identified as a risk factor for late onset Alzheimer’s disease (LOAD) in genome wide association studies [119,120]. *PICALM* is one of a small group of genes associated with LOAD that code for endocytic proteins. This group includes the *BIN1* gene, which codes for amphiphysin 2, an accessory protein with a CLAP domain. Which CALM functions impact on LOAD are not clear. The most significant single nucleotide polymorphism (SNP), rs3851179 [119,121], has an odds ratio (0.85 [119]) that indicates this SNP results in a protective allele. This correlates with a study showing improved episodic memory performance for the minor allele [122]. The rs3851179 SNP is in a non-coding region ~80 kb 5′ of *PICALM* with no known function. Less significant SNPs are located in regions that may be involved in transcription factor binding and exon splicing and may be in linkage disequilibrium with rs3851179 [119]. This suggests that the association between *PICALM* and LOAD may relate to changes in expression of CALM or particular CALM isoforms. Although much focus has been on this highly significant SNP, rare *PICALM* mutations might be found to have greater penetrance.

**Figure 4:**
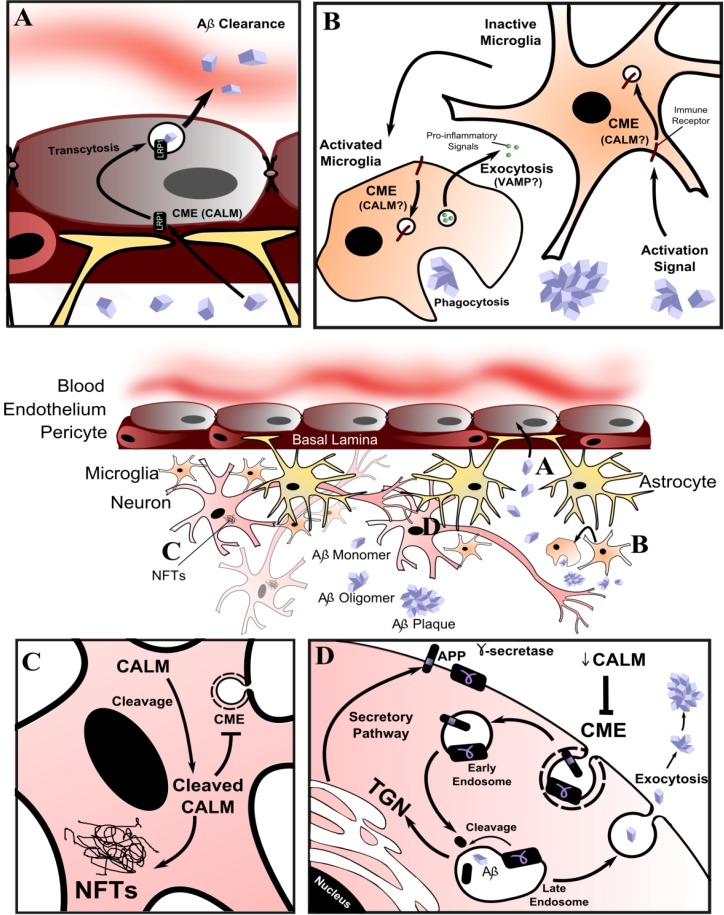
Current hypotheses of the mechanism of CALM involvement in late onset Alzheimer’s disease (LOAD). (**A**) CALM may influence the efficiency of Aβ clearance into the bloodstream [127,128,129], possibly by changing the LRP1 and CME dependent brain-to-blood transcytosis of Aβ; (**B**) CALM is highly expressed in microglia in individuals with LOAD [127]. CALM dysfunction in microglial cells of the innate immune system may affect immune system signaling and Aβ clearance; (**C**) CALM is cleaved in LOAD brain tissue and associated with hyper-phosphorylated tau in neurofibrillary tangles (NFTs) [127]; (**D**) CALM may be involved in Aβ generation by influencing amyloid precursor protein (APP) processing. Altered CALM function/expression could change the steady-state localization of APP and γ-secretase via CME and thus alter the rate of APP cleavage and production of Aβ. In this latter hypothesis, decreased CALM expression might be protective. Conflicting evidence relates the level of CALM expression to the production of Aβ [86,134,136].

A number of studies have examined *PICALM*/CALM expression. *PICALM* mRNA levels and LOAD associated SNPs have not correlated with LOAD pathology in brain tissue samples [123,124,125]. In support of a role for CALM, increased expression was found in the cortex of Tg2576 mice that overexpress the Swedish mutation in amyloid precursor protein (APP) [126]. Also, CALM was found to be cleaved in diseased brain tissue and associated with hyper-phosphorylated tau in neurofibrillary tangles [127] (Figure 4). Technical difficulties in handling and obtaining sufficient numbers of *post mortem* samples may contribute to the lack of a conclusive link to LOAD-associated SNPs.

A potential confounding factor is that CALM is expressed more highly in the microvasculature of the brain than neurons [127,128,129] and is highly expressed in microglia in individuals with LOAD [127]. These two observations have led to two hypotheses (Figure 4). 

First, CALM might contribute to LOAD in endothelial cells by changing the efficiency of clearance of amyloid-beta (Aβ) through the blood-brain-barrier [127,128,129]. Since Aβ likely enters endothelial cells via low-density lipoprotein receptor-related protein 1 (LRP1) [130] using CME [131], a change in CALM abundance or function would affect uptake and transcytosis to the bloodstream. Second, aberrant CALM function in the microglial cells of the innate immune system might result in reduced Aβ clearance or an inflammatory state that contributes to LOAD [127], perhaps by influencing the recycling of immune receptors or secretion of cytokines (via CME and VAMP dysregulation, respectively). Genes coding for proteins related to the immune system or expressed in microglia have also been associated with LOAD [132,133]. However, these hypotheses have not been tested and both imply that CALM contributes only after LOAD pathology has been established in neurons by another mechanism.

Knock-down of CALM was shown to affect the trans-Golgi network (TGN)/endosomal sorting system in non-neuronal cells [14], which has led to molecular and biochemical studies on how CALM influences APP processing [86,134] (Figure 4). APP is initially synthesized in the endoplasmic reticulum, modified in the Golgi and then transported to the cell surface by the secretory pathway. From there it is internalized back into the cell by endocytosis, trafficked from early endosomes to late endosomes or the TGN, before being proteolyzed to release toxic Aβ. The role of CALM in Aβ regulation has been studied in cellular models, yeast and mice [86,134]. CALM and APP were shown to co-localize during endocytosis in neuroblastoma N2a cells stably overexpressing APP. CALM knockdown reduced APP internalization and Aβ production, and overexpression reversed this effect [86]. A similar effect was found in hippocampal tissue from APPswe/PS1ΔE9 mice injected with viral vectors to knockdown/overexpress CALM. CALM overexpression resulted in increased amyloid plaque load [86]. In a screen of yeast genes, it was found YAP180B overexpression suppressed a model of Aβ toxicity [134]. Overexpressed Unc11 in glutamatergic neurons of *C. elegans* and CALM in cortical neurons also suppressed models of Aβ toxicity [134]. A recent study showed CALM depletion reduced production of toxic Aβ and this was because of a shift in CALM-dependent CME of γ-secretase, the enzyme responsible for APP cleavage [135]. Not all of these studies agree, but they demonstrate the potential for CALM to influence APP processing by changing the membrane steady-state localisation of APP and/or γ-secretase (Figure 4).

Alzheimer’s disease brains have reduced expression of AP180 and other SV cycle proteins and fewer synapses overall [136,137]. Knockdown of AP180, but not CALM, reduced Aβ generation in a neuronal cell line [138], contradicting other studies. Also, AP180 was identified as a causal regulator of LOAD in a large scale gene expression and network analysis of post mortem samples [133]. Although CALM is ubiquitously expressed and AP180 is brain-specific, the gene for AP180, *SNAP91*, has not been genetically linked to LOAD. However, *SNAP91* has been associated with mood-incongruent psychotic bipolar disorder [139] and, as with other SV cycle proteins, continues to be a potential therapeutic target for diseases related to aberrant neurotransmission.

## 9. Conclusions

The overall functions of the ANTH and AD of CALM and AP180 in lipid binding, VAMP sorting and clathrin assembly are well established. However, there is a lack of mechanistic detail and some unanswered questions about CALM and AP180 function. In what order do clathrin and AP2 interact with CALM and AP180? Many clathrin binding sites on AP180 and some for CALM have been identified, but what is the mechanism of assembly and how are uniform vesicles achieved? Approaches that allow an acute knockout/knockdown or single molecule resolution analysis of CALM and AP180 may provide this detail. Since the early days of AP180 discovery, a three domain structure has been postulated, and sequence similarity suggests the same domain organization for CALM. The function of this high pI C-terminal domain/sub-domain remains to be discovered. The VAMP sorting function is well established for CALM, AP180 and homologs in multiple organisms. However, there is an unexplained difference in VAMP affinity for CALM and AP180. The disruption of TGN/endosomal trafficking and receptor internalization when CALM is knocked down, might be a consequence of VAMP mis-localization and subsequent inability of vesicles to fuse with correct target membranes. This is not yet proven, but is perhaps the most likely way that APP processing might be affected by dysregulated CALM. The CALM functions and specific biological process involving CALM in LOAD remain to be determined. A greater understanding of the CALM mediated CME mechanisms, cell signaling via post-translational modifications and broader biological roles may lead to research tools and therapeutics for LOAD and diseases related to neurotransmission.
